# The prevalence and correlates of low sexual functioning in women on hemodialysis: A multinational, cross-sectional study

**DOI:** 10.1371/journal.pone.0179511

**Published:** 2017-06-20

**Authors:** Valeria Saglimbene, Patrizia Natale, Suetonia Palmer, Marco Scardapane, Jonathan C. Craig, Marinella Ruospo, Letizia Gargano, Giuseppe Lucisano, Marietta Török, Eduardo Celia, Rubén Gelfman, Anna Bednarek-Skublewska, Jan Dulawa, Paul Stroumza, Miguel Leal, Domingo Del Castillo, Angelo Marco Murgo, Staffan Schon, Charlotta Wollheim, Jörgen Hegbrant, Giovanni F. M. Strippoli

**Affiliations:** 1 Medical Scientific Office, Diaverum, Lund, Sweden; 2 Sydney School of Public Health, University of Sydney, Sydney, Australia; 3 Department of Medicine, University of Otago Christchurch, Christchurch, New Zealand; 4 Centre for Outcomes, Research and clinical Epidemiology, Pescara, Italy; 5 Division of Nephrology and Transplantation, Department of Translational Medicine, Amedeo Avogadro University of Eastern Piedmont, Novara, Italy; 6 Medical University of Lublin, Lublin, Poland; 7 SHS, Medical University of Silesia, Katowice, Poland; 8 Diaverum Academy, Bari, Italy; 9 Department of Emergency and Organ Transplantation, University of Bari, Bari, Italy; Hospital Universitario de la Princesa, SPAIN

## Abstract

Sexual dysfunction may affect 80% of women in hemodialysis. However the specific patterns and clinical correlates of sexual functioning remain poorly described. The aim of this study was to assess prevalence and correlates of the individual domains of sexual functioning in women treated with hemodialysis. We recruited, into this multinational cross-sectional study, women treated with long-term hemodialysis (Collaborative Working Group on Depression and Sexual dysfunction in Hemodialysis study). Self-reported domains of sexual functioning were assessed by the Female Sexual Function Index, which is routinely administered within the network of dialysis patients followed by the working group. Lower scores represented lower sexual functioning. Socio-demographic and clinical correlates of each domain of sexual functioning were identified by stepwise multivariable linear regression. Sensitivity analyses were restricted to women who reported being sexually active. We found that of 1309 enrolled women, 659 (50.3%) provided complete responses to FSFI survey questions and 232 (35%) reported being sexually active. Overall, most respondents reported either no sexual activity or low sexual functioning in all measured domains (orgasm 75.1%; arousal 64.0%; lubrication 63.3%; pain 60.7%; satisfaction 60.1%; sexual desire 58.0%). Respondents who were waitlisted for a kidney transplant reported scores with higher sexual functioning, while older respondents reported scores with lower functioning. The presence of depression was associated with worse lubrication and pain scores [mean difference for depressed versus non-depressed women (95% CI) -0.42 (-0.73 to -0.11), -0.53 (-0.89 to -0.16), respectively] while women who had experienced a previous cardiovascular event reported higher pain scores [-0.77 (-1.40- to -0.13)]. In conclusion, women in hemodialysis reported scores consistent with marked low sexual functioning across a range of domains; the low functioning appeared to be associated with comorbidity.

## Introduction

Despite significant advances in the management of end-stage kidney disease (ESKD) including renal replacement therapy and the increasing availability of newer and more effective pharmacological interventions, there has been no marked improvement in patient survival, which remains 3–4 years on average [1–7]. Depression, pain, pruritus, impaired sleep, and fatigue are commonly reported by people undergoing long-term hemodialysis [8–10]. In addition, sexual dysfunction is also very common in both men and women with ESKD [11], is associated with anxiety and depression, and may affect quality of life through effects on self-confidence, self-esteem and self-image [12–14].

However, previous research in this area has principally focused on erectile dysfunction in men, while sexual dysfunction among women on hemodialysis has received less attention [11]. Our Collaborative Working Group on Depression and Sexual dysfunction (CDS) in Hemodialysis has previously conducted a large scale study (involving 1472 women) to examine the prevalence and correlates of low self-reported sexual functioning in women treated with hemodialysis [15]. In the CDS study, four out of five women reported Female Sexual Function Index (FSFI) scores consistent with low sexual functioning (84%). Although this previous study showed a high prevalence of low sexual functioning in this clinical setting, an analysis of the individual domains of sexual functioning (desire, arousal, lubrication, overall satisfaction with sexual life and pain) has not been previously reported.

Exploring the characteristics of individual sexual functioning domains may contribute to an improved understanding of the specific sexual experiences of women, when treated with hemodialysis, to inform a patient-centered research agenda. In this study, we assessed the prevalence and correlates of the individual domains of sexual functioning in women treated with hemodialysis within the same cohort of patients involved in the CDS study.

## Material and methods

CDS is a multinational, cross-sectional study involving women with ESKD receiving long-term outpatient hemodialysis for renal replacement therapy [15]. In this report, we evaluated the prevalence and correlates of each sexual functioning dimension of the of Female Sexual Function Index (desire, arousal, lubrication, orgasm, satisfaction and pain) [16] in this population. The study has been reported according to the Strengthening the Reporting of Observational studies in Epidemiology (STROBE) guidelines [17].

### Study population

We included consecutive women aged 18 years or older who had been treated with long-term hemodialysis for any duration within a convenience sample of clinics located in Europe (France, Hungary, Italy, Poland) and South America (Argentina). The clinics were part of a collaborative dialysis network coordinated by Diaverum and were from communities and countries for which local investigators were highly committed to routine administration of a series of questionnaires and diagnostic instruments relating to depression, quality of life and sexual dysfunctions to patients within a selected period of time. We enrolled participants between January and June 2008. We excluded women who had major psychiatric disorder or who declined to participate in the study. Women were enrolled after having provided written informed consent to give information on sexual dysfunction, satisfaction and depression. We obtained ethics approval for analysis of routinely collected data from the University of Sydney Human Research Ethics Committee (project number for approval 2013/031). The study was conducted according to the principles of the Declaration of Helsinki.

### Data collection

Women were administered the Female Sexual Function Index [16] survey during a standard hemodialysis treatment. The Female Sexual Function Index evaluates self-reported sexual functioning during the previous month and includes 19 items grouped within six central domains: desire (items 1 and 2), arousal (items 3 to 6), lubrication (items 7 to 10), orgasm (items 11 to 13), global sexual and relationship satisfaction (items 14 to 16), and pain (items 17 to 19). Each domain was scored on a scale of 0 to 6 with lower scores indicating lower sexual functioning. A domain score of 0 indicated that the women reported no sexual activity. The individual domain scores were then totaled and multiplied by a predetermined factor to weight each domain equally (S1 Table). Depression symptoms were concurrently evaluated using the Center for Epidemiologic Studies-Depression (CESD) instrument [18]. A score ≥18 was compatible with depression [19]. We used questionnaires in the participant’s native language after surveys underwent translation and linguistic validation by the MAPI Institute http://mapigroup.com.

Questionnaires were completed anonymously. Demographic, clinical, laboratory and dialysis-related data were obtained from a centralized database linked using a unique identification code. Standardized clinical variables included age, gender, country of treatment, education level, presence of partner, occupational status, smoking history, physical activity, being on the waiting list for a kidney transplant, comorbidities including previous cardiovascular disease and diabetes, medication use, serum parameters including hemoglobin, phosphorus, parathyroid hormone, calcium and albumin, and dialysis characteristics.

### Statistical analyses

Baseline socio-demographic, clinical and dialysis-related characteristics were summarized as mean (standard deviation) or median (25th to 75th percentile) for continuous variables and frequencies and percentages for categorical variables. Prevalence of each domain of low female sexual functioning was reported as median (25th to 75th percentile). Frequency and percentages of each item of the FSFI have been also calculated.

Women were classified as respondents when they answered all of the 19 questions of the FSFI questionnaire, incomplete respondents when one or more answers were missing. The clinical and socio-demographic characteristics of respondents and incomplete respondents were compared using standard univariate methods.

For each domain, we conducted a stepwise multivariable linear regression to identify correlates of low or high scores controlling for the following covariates judged to have clinical importance: age, depression symptoms (CES-D score ≥ 18), pregnancy, occupational and menopause status, experience of a prior cardiovascular event (including myocardial infarction, stroke or transient ischemic attack, or coronary or other revascularization surgery as assessed by the treating physician), neurologic conditions (spinal cord lesions, multiple sclerosis, Parkinson disease, or Alzheimer disease), previous kidney transplant, wait-listing for kidney transplant, anxiolytics medication, time on dialysis, mean arterial pressure and serum phosphorus. We used α = 0.1 as a threshold for a covariate to both enter and exit the final model. Results were expressed as mean differences in domain scores along with their 95% confidence intervals (95% CIs). Finally, we performed a sensitivity analysis in those women who reported being sexually active.

We considered two-sided P-value <0.05 as statistically significant. All analyses were performed using SAS Statistical Package Release 9.3 (SAS Institute, Cary, NC, USA).

## Results

### Participants characteristics

**Fig 1:**
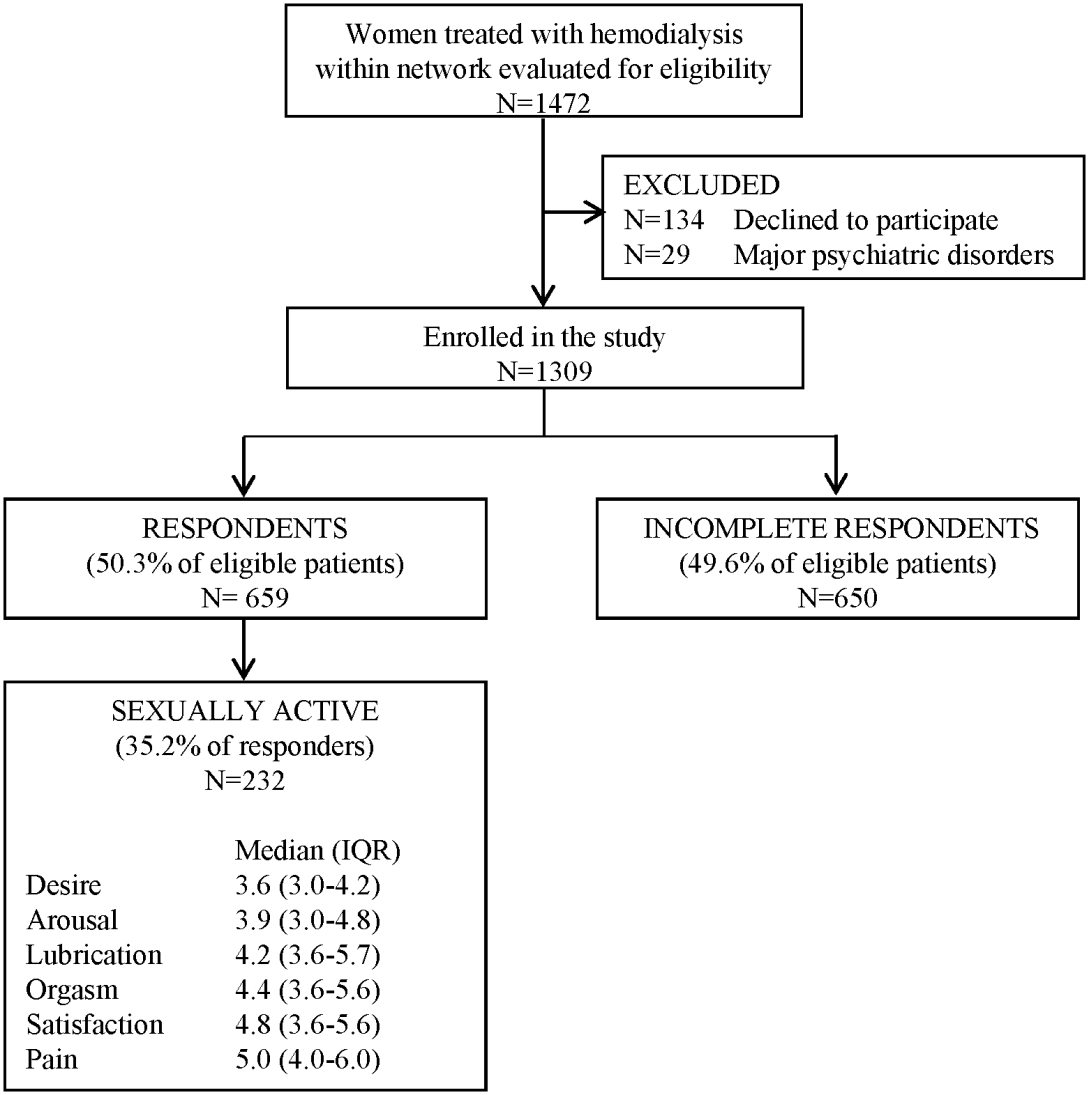
Flow chart showing identification of study participants.

Among 1472 consecutive women assessed for eligibility, 163 were excluded: 134 declined to respond to the routinely administered questionnaires, and 29 had a concurrent major psychiatric disorder precluding participation. The remaining 1309 (88.9%) received the FSFI questionnaire. 659 (50.3%) answered all of the 19 questions and were included in these analyses (Fig 1). The percentage of missing answers was very similar across all questions. Patients were likely to answer all questions or none or almost none. Compared with respondents, incomplete respondents were older (66.8±14.5 years versus 58.8±15.3 years; P <0.001), living without a partner (54.6% versus 44.2%; P <0.001), post-menopausal (76.2% versus 62.2%; P <0.001) and were receiving shorter dialysis treatment (228.4±20.5 minutes versus 233.5±23.9; P<0.001). Table 1 displays the baseline characteristics of the overall sample, survey respondents and incomplete respondents. The raw study data are provided in Tables A and B in S1 File.

**Table 1:** Socio-demographic, clinical and dialysis related characteristics of women who responded and those who did not respond to FSFI questionnaire *.

Characteristic	Overall (n = 1309)	Respondents (n = 659)	Incomplete respondents (n = 650)	P value^≠^
**Age** (year)	62.8±15.4	58.8±15.3	66.8±14.5	<0.001
**Highest school education**, n (%)				0.3
≤5 years	605 (46.2)	304 (46.1)	301 (46.3)	
5–8 years	456 (34.8)	233 (35.4)	223 (34.3)	
>8years	177 (13.5)	90 (13.6)	87 (13.4)	
**Depression score** (CES-D scale)	20.8±11.3	20.7±11.3	20.9±11.3	0.8
**Country**, n (%)				
Italy	428 (32.7)	286 (44.0)	142 (21.6)	<0.001
Hungary	327 (25.0)	75 (11.5)	252 (38.2)	
Argentina	285 (21.8)	79 (12.2)	206 (31.3)	
Poland	215 (16.4)	166 (25.5)	49 (7.4)	
France	54 (4.1)	44 (6.8)	10 (1.5)	
**Living without partner**, n (%)	633 (49.3)	287 (44.2)	346 (54.6)	<0.001
**Waiting list for kidney transplant**, n (%)	159 (12.1)	104 (15.8)	55 (8.5)	<0.001
**Occupational status**, n (%)				0.002
Employed	106 (8.1)	60 (9.1)	46 (7.1)	
Unemployed	224 (17.1)	131 (19.9)	93 (14.3)	
Receiving pension	958 (73.2)	453 (68.7)	505 (77.7)	
**Previously had children**, n (%)	975 (74.5)	504 (76.5)	471 (72.5)	0.002
**Menopause**, n (%)	905 (69.1)	410 (62.2)	495 (76.2)	<0.001
**Comorbid condition**, n (%)				
Diabetes mellitus	295 (22.5)	144 (21.9)	151 (23.2)	0.7
Hypertension	786 (60.0)	402 (61.0)	384 (59.1)	0.8
Prior cardiovascular event^†^	103 (7.9)	49 (7.4)	54 (8.3)	0.6
Kidney transplant	35 (45.5)	25 (48.1)	10 (40.0)	0.5
**Primary renal disease**, n (%)				0.1
Diabetic nephropathy	189 (14.7)	111 (17.2)	78 (12.2)	
Hypertensive nephrosclerosis	287 (22.4)	137 (21.2)	150 (23.5)	
Other	808 (63.0)	398 (61.5)	410 (64.2)	
**Current or former smoker**, n (%)	223 (17)	151 (22.9)	72 (11.1)	<0.001
**Clinical characteristics**				
Interdialytic weight gain (kg)	2.1±0.9	2.0±0.9	2.1±0.9	0.02
Time on dialysis (months)	41.8 (18.3–76.8)	40.0 (17.0–77.7)	43.6 (20.3–75.4)	0.6
Duration of dialysis (min/session)	231.0±22.4	233.5±23.9	228.4±20.5	<0.001
Single pool Kt/V	1.6±0.3	1.6±0.3	1.6±0.3	0.3
Systolic blood pressure (mmHg)	128.6±18.8	130.2±19.2	127.0±18.3	0.02
Hemoglobin (g/dL)	10.9±1.3	10.9±1.3	10.9±1.3	0.7
Serum ferritin (μg/L)	420.0 (239.0–660.0)	454.5 (276.0–686.0)	375.0 (211.5–609.5)	<0.001
Serum albumin (g/dL)	3.8±0.4	3.9±0.4	3.8±0.5	0.004
LDL cholesterol (mg/dL)	101.3±34.7	100.2±37.8	102.0±32.6	0.2
**Medication** (%)				
Beta blocker	473 (36.1)	257 (39.0)	216 (33.2)	0.03
ACE inhibitor	383 (29.3)	217 (32.9)	166 (25.5)	0.003
Angiotensin receptor blocker	156 (11.9)	89 (13.5)	67 (10.3)	0.07
Erythropoietin	1192 (91.1)	601 (91.2)	591 (90.9)	0.9
Lipid lowering therapy	381 (29.1)	193 (29.3)	188 (28.9)	0.9
Antidepressant	86 (6.6)	42 (6.4)	44 (6.8)	0.8
Antipsychotic	42 (3.2)	14 (2.1)	28 (4.3)	0.03
Anxiolytic	241 (18.4)	153 (23.2)	88 (13.5)	<0.001

*Data expressed with a plus/minus sign were mean ± SD. Medians were expressed with interquartile range. Numbers may not sum to group totals or percentages may not total 100% where data for the variable are missing. ACE, angiotensin-converting enzyme; CES-D, Center for Epidemiological Studies-Depression; LDL, low density lipoprotein

^≠^P value for comparison between who responded and those who did not respond to FSFI questionnaire

^†^Prior cardiovascular event included myocardial infarction, stroke or transient ischemic attack, or coronary or other revascularization surgery as assessed by the treating physician

### Prevalence of domains of female sexual functioning

**Fig 2:**
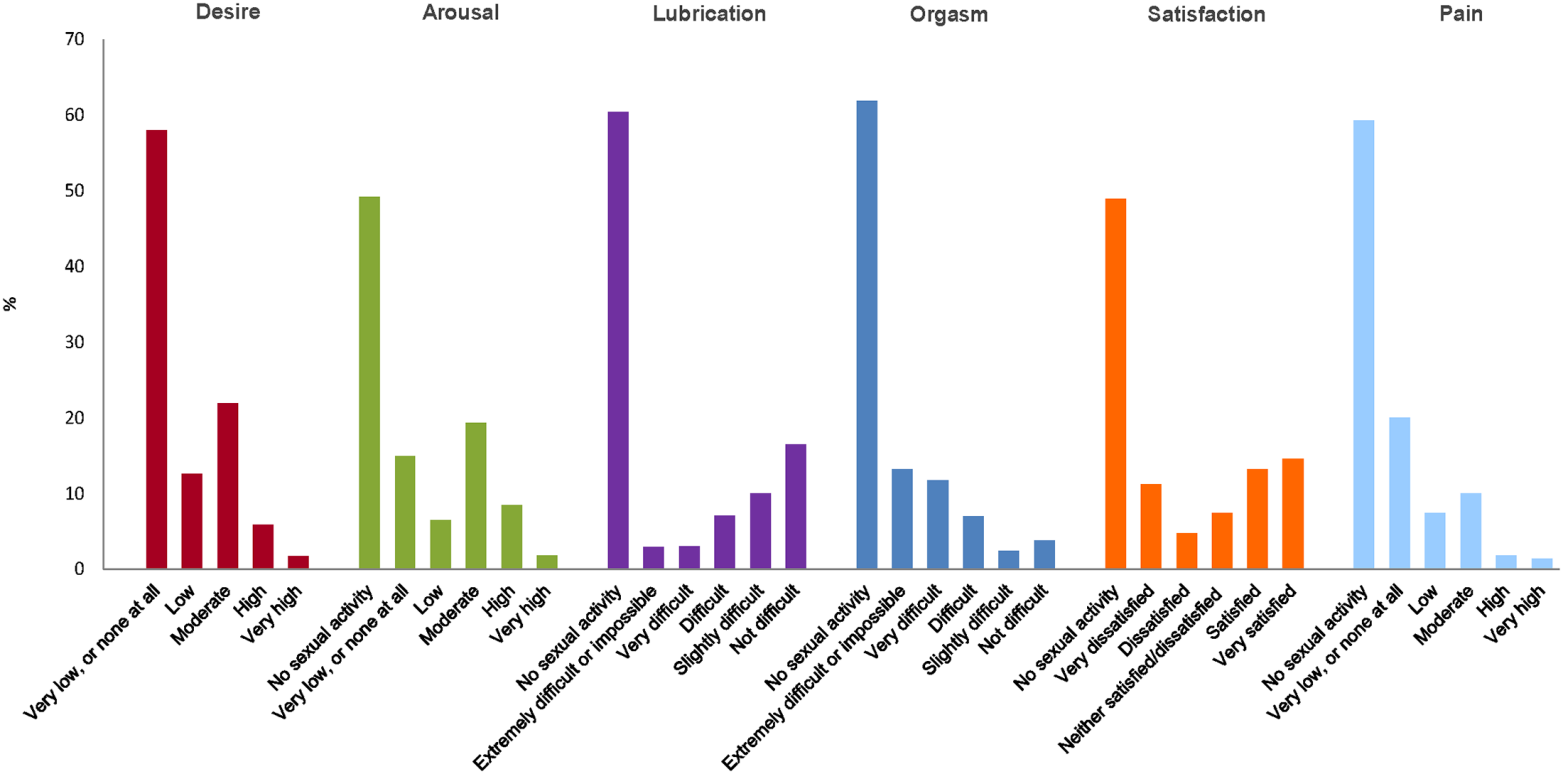
Prevalence of sexual problems in women who responded to the FSFI questionnaire (N = 659).

Fig 2 illustrates the sexual functioning by domain in respondents. For the desire domain, 382 respondents (58.0%) reported very low or no sexual desire. For the arousal domain, 324 respondents (49.2%) had no sexual activity and 98 (14.9%) reported very low or no sexual arousal. In the lubrication domain, 398 respondents (60.4%) reported no sexual activity and 19 (2.9%) reported that becoming lubricated was extremely difficult or impossible. In the orgasm domain, 408 respondents (61.9%) reported no sexual activity and 87 respondents (13.2%) reported scores consistent with extreme difficulty or inability to reach orgasm. In the satisfaction domain, 322 respondents (48.9%) reported no sexual activity and 74 (11.2%) reported scores indicating they were very dissatisfied with their overall sexual and life. In the pain domain, 391 respondents (59.3%) reported no sexual activity while 9 (1.4%) reported very high pain during sexual activity. Overall, median scores (25^th^, 75^th^ percentile) were (lower scores indicating higher difficulty): desire 1.2 (1.2 to 3), arousal 0.6 (0 to 3.3), lubrication 0 (0 to 3.6), orgasm 0 (0 to 3.6), satisfaction 2.4 (0 to 4.4), pain 0 (0 to 4.4).

### Correlates of domains of female sexual functioning

Table 2 summarizes correlates of each domain of sexual functioning adjusted for socio-demographic and clinical variables.

**Table 2:** Correlates of individual domains of the Female Sexual Function Index (N = 659), displayed as multivariate adjusted mean difference *.

Correlates	Desire Mean change (95% CI)	Arousal Mean change (95% CI)	Lubrication Mean change (95% CI)	Orgasm Mean change (95% CI)	Satisfaction Mean change (95% CI)	Pain Mean change (95% CI)
**Age, per year increase**	-0.05 (-0.05 to -0.04)	-0.05 (-0.06 to -0.05)	-0.07 (-0.08 to -0.05)	-0.06 (-0.07 to -0.05)	-0.05 (-0.06 to -0.04)	-0.06 (-0.08 to -0.05)
**Wait list for transplant**	0.45 (0.21 to 0.70)	0.73 (0.38 to 1.08)	0.71 (0.30 to 1.13)	0.75 (0.33 to 1.17)	0.58 (0.15 to 1.00)	0.86 (0.37 to 1.34)
**Occupation**						
Employed	-	1.00	-	1.00	-	-
Retired	-	-0.49 (-0.93 to -0.04)	-	-0.63 (-1.17 to -0.09)	-	-
Unemployed	-	-0.11 (-0.58 to 0.36)	-	-0.20 (-0.77 to 0.36)	-	-
**Depression (CESD score ≥18)**	-	-	-0.42 (-0.73 to -0.11)	-	-	-0.53 (-0.89 to -0.16)
**Prior cardiovascular event**		-	-	-	-	-0.77 (-1.40 to -0.13)

*The multivariate model included age, depression symptoms (CES-D score ≥ 18), pregnancy, occupational and menopause status, experience of a prior cardiovascular event (including myocardial infarction, stroke or transient ischemic attack, or coronary or other revascularization surgery as assessed by the treating physician), neurologic conditions (spinal cord lesions, multiple sclerosis, Parkinson disease, or Alzheimer disease), previous kidney transplant, wait-listing for kidney transplant, anxiolytics medication, time on dialysis, mean arterial pressure and serum phosphorus

#### Desire

For each year increase in age, the desire score was reduced by -0.05 (95% CI -0.05 to -0.04)]. While women waitlisted for a kidney transplant [0.45 (0.21 to 0.70)] reported higher scores.

#### Arousal

Women waitlisted for a kidney transplant reported higher arousal scores [0.73 (0.38 to 1.08)]. Older women [-0.05 (-0.06 to -0.05)] and those who were retired [-0.49 (-0.93- to -0.04)] reported lower scores.

#### Lubrication

Women waitlisted for a kidney transplant [0.71(0.30 to 1.13)] reported higher lubrication scores. Increasing age [-0.44 (-0.82 to -0.06)] and the presence of depression [-0.66 (-0.97 to -0.35)] correlated with lower scores.

#### Orgasm

Women waitlisted for a kidney transplant reported higher orgasm scores [0.75 (0.33 to 1.17)]. Older women [-0.06 (-0.07 to -0.05)] and women who were retired [-0.63 (-1.17—to -0.09)] reported lower scores.

#### Satisfaction

Increasing age [-0.05 (-0.06 to -0.04)] was associated with reported lower satisfaction with overall sexual and relationship experiences. Women waitlisted for a kidney transplant [0.58 (0.15 to 1.00)] reported higher satisfaction.

#### Pain

Women waitlisted for a kidney transplant [0.86 (0.37 to 1.34)] experienced lower pain during sexual activity. Increasing age [-0.06 (-0.08 to -0.05)] and depression [-0.37 (-0.72 to -0.01)] or the experience of a prior cardiovascular event [-0.77 (-1.40 to -0.13)] were correlated with higher pain.

### Sensitivity analysis

Domain scores were influenced by sexual activity in the FSFI questionnaire (a score of 0 indicated no sexual activity). We therefore assessed prevalence and correlates of each domain of sexual dysfunction in a sensitivity analysis restricted to sexually active women. Among responders, 232 (35.2%) women reported being sexual active in all domains and could be included in sensitivity analyses. Median scores of each domain of sexual dysfunction in these women were: desire 3.6 (3.0 to 4.2), arousal 3.9 (3.0 to 4.8), lubrication 4.2 (3.6 to 5.7), orgasm 4.4 (3.6 to 5.6), satisfaction 4.8 (3.6 to 5.6) and pain 5.0 (4.0 to 6.0). Respectively, 7.8% and 19% had very low/no and low desire; 7.8% and 12.5% had very low/no and low arousal; 4.7% and 6.9% reported that becoming lubricated was extremely difficult/impossible and very difficult; 36.2% and 31.9% reported that it was extremely difficult/impossible and very difficult to reach an orgasm; 9.5% and 10.3% were very dissatisfied and dissatisfied with sexual life; 3.0% and 3.9% had very high and high pain (S1 Fig).

Table 3 summarizes the correlates of each domain of sexual dysfunction adjusted for socio-demographic and clinical variables in this sensitivity analysis. Multivariate analysis showed that the increasing age correlated with lower scores in each sexual dysfunction domain (except for pain) in sexually active women similarly to the overall respondent population. Wait-listing for kidney transplant was no longer correlate of most domains in sexually active women compared with the overall respondent population in this sensitivity analysis. The presence of depression symptoms (CESD score ≥18) was associated with lower arousal, lubrication, orgasm and higher pain.

**Table 3:** Correlates of individual domains of the Female Sexual Function Index in women who reported being sexually active (N = 232), displayed as multivariate adjusted mean difference *.

Correlates	Desire Mean change (95% CI)	Arousal Mean change (95% CI)	Lubrication Mean change (95% CI)	Orgasm Mean change (95% CI)	Satisfaction Mean change (95% CI)	Pain Mean change (95% CI)
**Age, per year increase**	-0.03 (-0.04 to -0.02)	-0.04 (-0.05 to -0.03)	-0.03 (-0.04 to -0.02)	-0.02 (-0.03 to -0.01)	-0.03 (-0.04 to -0.01)	
**Wait list for transplant**	-	-	-	-	-	0.41 (0.08 to 0.74)
**Depression (CES-D score ≥18)**	-	-0.53 (-0.83 to -0.22)	-0.84 (-1.16 to -0.52)	-0.46 (-0.79 to -0.14)	-	-1.08 (-1.40 to -0.76)

*The multivariate model included age, depression symptoms (CES-D score ≥ 18), pregnancy, occupational and menopause status, experience of a prior cardiovascular event (including myocardial infarction, stroke or transient ischemic attack, or coronary or other revascularization surgery as assessed by the treating physician), neurologic conditions (spinal cord lesions, multiple sclerosis, Parkinson disease, or Alzheimer disease), previous kidney transplant, wait-listing for kidney transplant, anxiolytics medication, time on dialysis, mean arterial pressure and serum phosphorus

## Discussion

In this cross-sectional study of 1309 women treated with hemodialysis, approximately half anonymously provided information about sexual function. Of these 659 women, 427 reported no sexual activity and 232 reported marked low sexual function across all measured domains including desire, arousal, lubrication, orgasm, satisfaction, and pain. Women waitlisted for a kidney transplant reported higher scores in all sexual domains, while older women reported lower scores. The presence of depression was associated with lower lubrication and higher pain scores; while women who had experienced a cardiovascular event also reported a higher pain score on average. The high prevalence of sexual issues and correlated characteristics were confirmed in the subgroup of women who reported being sexual active. These data suggest sexual function in women treated with dialysis is frequently very poor and may warrant further investigation to understand the clinical relevance of the data from a patient perspective. In our study women on hemodialysis didn’t report very high levels of dissatisfaction against their severe sexual problems. This may be consistent with the low sexual desire they reported that could show lack of interest in sexual activity as part of their life. Previous studies have already explored patients views regarding research priorities in dialysis setting [20, 21] but did not include sexual function. Additional qualitative studies exploring patient experiences and beliefs might assist greater understanding of the impact of sexual dysfunction on the overall quality of life in this population [22].

We performed a comprehensive evaluation of all individual domains of sexual function in women with end-stage kidney disease in a relatively large study. We assessed the prevalence and correlates of each domain adjusted for socio-demographic and clinical variables. Previous studies reporting individual domains of female sexual function in women on hemodialysis had smaller sample sizes and used a range of different tools for assessment [23–28]. Five previous observational studies have evaluated potential associations for each domain, but only one of these performed an adjusted analysis for demographic and clinical characteristics [24–28]. Older age, hypertriglyceridemia and higher scores on the Beck Depression Inventory (BDI) scale for assessment of depression symptoms were associated with lower scores in each sexual dimension of women on hemodialysis [26]. Similarly in our study we found that older women had lower scores in each domain of the FSFI (indicating low functioning), while depression symptoms correlated with worse scores for lubrication and pain in the overall population and among sexually active women. Older age and presence of depression are correlates of lower sexual functioning also in healthy women [29, 30]. In particular, age is negatively correlated with scores of sexual functioning across most domains also in the general population [31, 32]. Consistently with our finding in hemodialysis, a recent study in healthy women found a strong association between sexual pain, vaginal dryness and depressive symptoms [33].

The strengths of our study are the inclusion of several hemodialysis centers within multiple countries, and the involvement of a sufficiently large number of women to facilitate adjustment for clinically relevant confounding variables.

However, our study has limitations which need to be considered when interpreting the findings. First, the response rate of 50.3% may have introduced selection bias. While low, this response rate is similar or better than that of other patient survey studies of sexual health [11, 26, 34, 35]. Second, women who did not respond to the survey were different from those who did, including being older, less likely to have a partner, and were more likely to be menopausal. Older women might have been less willing to discuss sexual issues with healthcare providers, even under the assurance of anonymity, although they may be equally or more likely to experience sexual problems compared with their younger peers. Third, the study did not measure the clinical distress that is a main diagnostic criterion for DSM-based definition of sexual dysfunction. Fourth, the cross-sectional design precluded understanding of the persistence of sexual problems in our population over time. In addition, the study could not draw causal inferences between sexual function and the potentially contributing factors we identified including depressive symptoms. Finally, observational studies of this nature are inherently prone to residual confounding and this is likely to explain the observation that being wait-listed for a kidney transplant correlated with sexual dysfunction independently of other factors, and probably reflected overall well-being.

Our study demonstrated a high prevalence of poor sexual functioning across all domains in women treated with hemodialysis. Despite a high frequency, treatment options for sexual dysfunction have not been adequately explored in this population [5]. Our data suggest that further quantitative and qualitative studies are required to understand whether existing questionnaires measure sexual problems that are relevant to patients in this population and to evaluate the impact of overall sexual dysfunction and its individual domains on patient quality of life. Collaboration between patients and clinicians is needed to identify whether sexual dysfunction is a research priority for women treated with hemodialysis. Physicians and researchers should be aware of the impact of poor sexual functioning in female dialysis patients to determine whether exploring new validated screening tools and intervention strategies for global or selected domain of sexual functioning is warranted.

In conclusion, sexual function scores are severely impaired in women with end-stage kidney disease and appear to be associated with comorbidity. Additional patient-centered studies might assist greater understanding of the impact of sexual dysfunction on quality of life in this population.

## Supporting information

S1 Table Female Sexual Function Index domain scores. (PDF)

S1 Fig Prevalence of sexual problems in women who reported being sexually active (N = 232). (PDF)

S1 File Raw study data. Table A. Baseline characteristics, Female Sexual Function Index and Center for Epidemiologic Studies-Depression (CESD) instrument. Table B. Legend.
(ZIP)
