# Supplementary material for: The prevalence and correlates of low sexual functioning in women on hemodialysis: A multinational, cross-sectional study
Source: PLoS One. 2017 Jun 20;12(6):e0179511. doi: 10.1371/journal.pone.0179511 (PMC5478101; doi:10.1371/journal.pone.0179511)
Supplement: S1 Table — (PDF) [file pone.0179511.s001.pdf]

S1 Table. Female Sexual Function Index domain scores

Domain	Items no.	Score Range	Factor	Minimum Score	Maximum Score
Desire	1, 2	1 – 5	0.6	1.2	6.0
Arousal	3, 4, 5, 6	0 – 5	0.3	0	6.0
Lubrication	7, 8, 9,10	0 – 5	0.3	0	6.0
Orgasm	11, 12, 13	0 – 5	0.4	0	6.0
Satisfaction	14, 15, 16	0– 5	0.4	0.8	6.0
Pain	17, 18, 19	0 – 5	0.4	0	6.0
