# Supplementary figures and images for: The prevalence and correlates of low sexual functioning in women on hemodialysis: A multinational, cross-sectional study
Source: PLoS One. 2017 Jun 20;12(6):e0179511. doi: 10.1371/journal.pone.0179511 (PMC5478101; doi:10.1371/journal.pone.0179511)

**S1 Fig. Prevalence of sexual problems in women who reported being sexually active (N=232)**

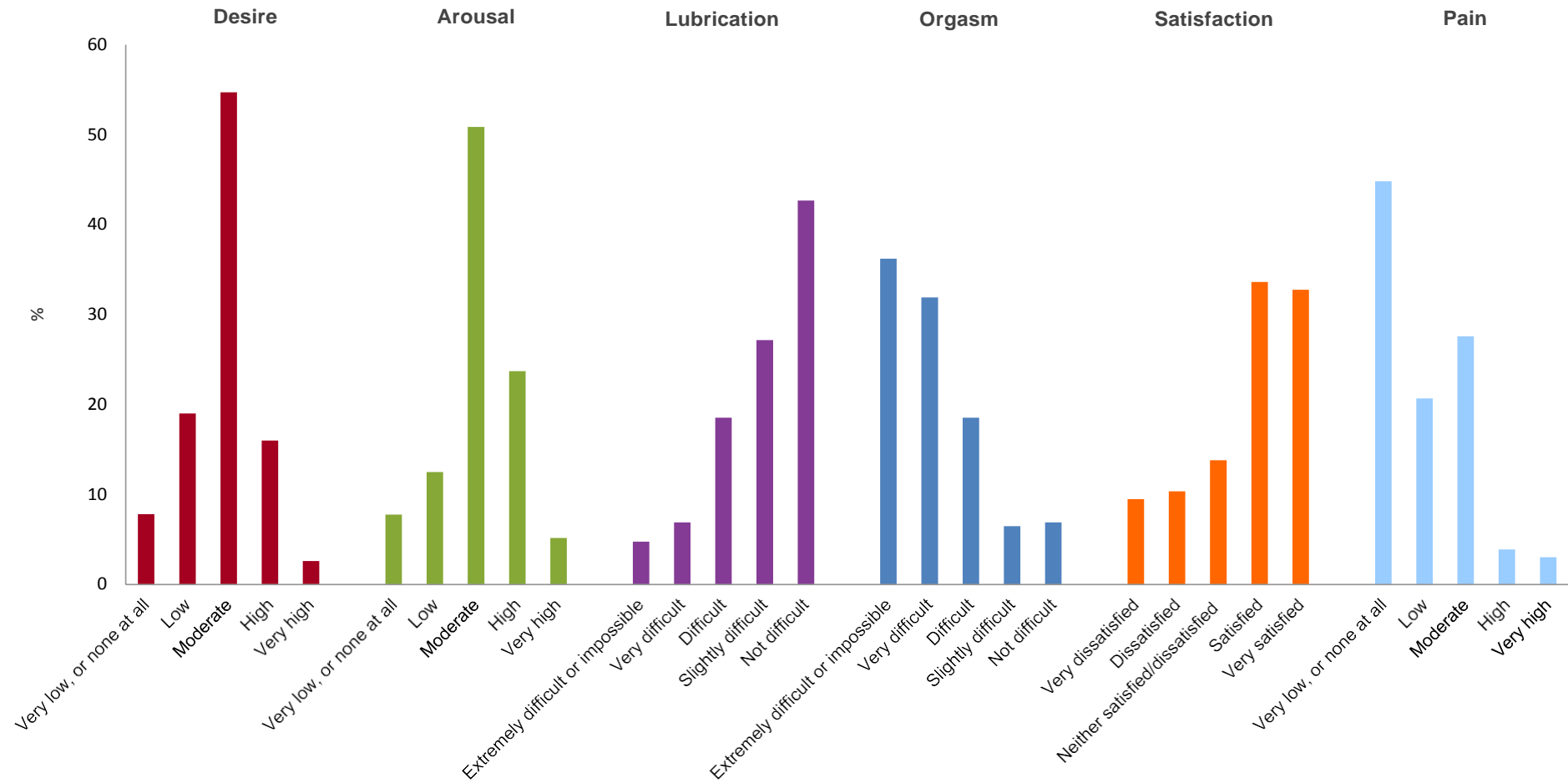

Supplement: S1 Fig — (PDF) [file pone.0179511.s002.pdf]
